# Reduced GFAP Expression in Bergmann Glial Cells in the Cerebellum of Sigma-1 Receptor Knockout Mice Determines the Neurobehavioral Outcomes after Traumatic Brain Injury

**DOI:** 10.3390/ijms222111611

**Published:** 2021-10-27

**Authors:** Gundega Stelfa, Edijs Vavers, Baiba Svalbe, Rinalds Serzants, Anna Miteniece, Lasma Lauberte, Solveiga Grinberga, Baiba Gukalova, Maija Dambrova, Liga Zvejniece

**Affiliations:** 1 Laboratory of Pharmaceutical Pharmacology, Latvian Institute of Organic Synthesis, Aizkraukles Str. 21, LV-1006 Riga, Latvia; edijs.vavers@farm.osi.lv (E.V.); baibas@farm.osi.lv (B.S.); rinalds.serzants@farm.osi.lv (R.S.); anna.miteniece18@imperial.ac.uk (A.M.); lasma@farm.osi.lv (L.L.); maija.dambrova@farm.osi.lv (M.D.); liga@farm.osi.lv (L.Z.); 2 Faculty of Veterinary Medicine, Latvia University of Life Sciences and Technologies, K Helmana Str. 8, LV-3001 Jelgava, Latvia; 3 Department of Pharmaceutical Chemistry, Riga Stradins University, Dzirciema Str. 16, LV-1007 Riga, Latvia; 4 Department of Life Sciences, Imperial College London, Exhibition Road, London SW7 2AZ, UK; 5 Laboratory of Physical Organic Chemistry, Latvian Institute of Organic Synthesis, Aizkraukles Str. 21, LV-1006 Riga, Latvia; solveiga@osi.lv (S.G.); baiba.gukalova@osi.lv (B.G.)

**Keywords:** sigma-1 receptor, traumatic brain injury, lateral fluid percussion injury, neurobehavior, cerebellum, astrocytes

## Abstract

Neuroprotective effects of Sigma-1 receptor (S1R) ligands have been observed in multiple animal models of neurodegenerative diseases. Traumatic brain injury (TBI)-related neurodegeneration can induce long-lasting physical, cognitive, and behavioral disabilities. The aim of our study was to evaluate the role of S1R in the development of neurological deficits after TBI. Adult male wild-type CD-1 (WT) and S1R knockout (S1R-/-) mice were subjected to lateral fluid percussion injury, and behavioral and histological outcomes were assessed for up to 12 months postinjury. Neurological deficits and motor coordination impairment were less pronounced in S1R-/- mice with TBI than in WT mice with TBI 24 h after injury. TBI-induced short-term memory impairments were present in WT but not S1R-/- mice 7 months after injury. Compared to WT animals, S1R-/- mice exhibited better motor coordination and less pronounced despair behavior for up to 12 months postinjury. TBI induced astrocyte activation in the cortex of WT but not S1R-/- mice. S1R-/- mice presented a significantly reduced GFAP expression in Bergmann glial cells in the molecular layer of the cerebellum compared to WT mice. Our findings suggest that S1R deficiency reduces TBI-induced motor coordination impairments by reducing GFAP expression in Bergmann glial cells in the cerebellum.

## 1. Introduction

Traumatic brain injury (TBI) is a complex neurodegenerative condition that is induced by biomechanical forces applied to the brain [1]. TBI is the main injury-related cause of permanent disability, with more than 50 million individuals suffering from TBIs each year [2]. Brain injury is commonly viewed as an acute self-limiting problem; however, the consequences of adult brain injuries can develop over years or even decades after the initial insult [3,4]. TBI is considered a “biphasic injury” that is characterized by direct physical and irreversible primary damage to the brain tissue and delayed secondary injury, which can be prevented and reduced by therapeutic intervention [5]. In addition, the progressive, long-lasting consequences of TBI have received increasing attention in both experimental and clinical studies [6,7,8].

Among the putative targets of neuroprotective drugs, the Sigma-1 receptor (S1R) has attracted increasing attention as a novel molecular target for treating neurological disorders [9]. S1R is a unique endoplasmic reticulum protein that is widely expressed in multiple organs, including the central nervous system [10]. S1R has been reported to play a role in both neurodegenerative and ischemic diseases, including Alzheimer’s disease, Parkinson’s disease, amyotrophic lateral sclerosis, stroke, and TBI [9]. Genetic inactivation and pharmacological inhibition of S1R are associated with neurodegenerative phenotypes [9,11,12]. Specific agonists of S1R have previously been shown to provide potent neuroprotection by attenuating neurodegeneration and preventing microglial cell activation in adult animal models of TBI and in neonatal hypoxic/ischemic brain damage [13,14]. However, S1R antagonists also exert neuroprotective effects on experimental models of brain ischemia. For example, mice treated with the antagonist S1RA presented significantly reduced cerebral infarct size and neurological deficits caused by permanent middle cerebral artery occlusion (MCAO) [15]. Additionally, the nonselective S1R antagonist haloperidol induces neuroprotection after brain ischemia [16]. Interestingly, after genetic inactivation and pharmacological blockade of S1R, antinociceptive effects on mice with traumatic spinal cord injury (SCI) were detected [17]. On the other hand, activation of S1R after SCI is presumed to be detrimental for neuron survival and motor function recovery [18]. Thus, different S1R ligands may exert various effects in a variety of neurodegenerative disease models.

The aim of this study was to investigate behavioral and histological outcomes in an S1R knockout (S1R-/-) mouse model of lateral fluid percussion injury over a 12-month period. We also evaluated posttraumatic behavioral and histological outcomes of pharmacological blockade of S1R using the S1R antagonist BD-1063.

## 2. Results

### 2.1. S1R-/- Mice Exhibit Improved Acute Sensorimotor Ability and Motor Coordination following TBI

**Figure 1:**
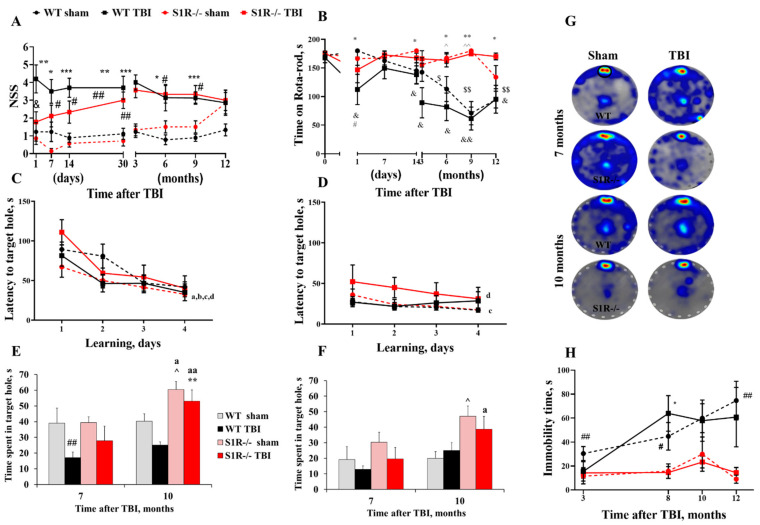
Long-term behavioral changes after traumatic brain injury (TBI). Behavioral testing revealed functional deficits in the (**A**) neurological severity score (NSS), (**B**) rota-rod (RR), (**C**–**G**) Barnes maze (BM), and (**H**) tail suspension tests. (**A**) Wild-type (WT) TBI mice showed neurological deficits compared to WT sham mice 24 h after injury. WT sham *n* = 9, WT TBI *n* = 7–10, Sigma-1 receptor knockout (S1R-/-) sham *n* = 6–8, S1R-/- TBI *n* = 6–9. * *p* < 0.05 WT TBI vs. S1R-/- TBI, # *p* < 0.05, ## *p* < 0.01 sham vs. TBI (the black symbols represent differences between WT groups, and the red symbols represent differences between S1R-/- groups). (**B**) WT TBI mice spent less time on the RR than WT sham mice 24 h after injury. Motor coordination was impaired in the WT TBI group at all time points after injury compared to baseline values. Motor coordination remained unaffected in S1R-/- mice after TBI. WT sham *n* = 8, WT TBI *n* = 6–9, S1R-/- sham *n* = 7, S1R-/- TBI *n* = 6–9. * *p* < 0.05, ** *p* < 0.01, *** *p* < 0.001 WT TBI vs. S1R-/- TBI, ^ *p* < 0.05, ^^ *p* < 0.01 WT sham vs. S1R-/- sham, ## *p* < 0.01 WT sham vs. WT TBI, $ *p* < 0.05, $$ *p* < 0.01 WT sham vs. baseline, & *p <* 0.05, && *p* < 0.01 WT TBI vs. baseline. In terms of BM performance, (**C**) all groups showed a reduced latency to find the target hole, indicating no impairments in the learning task (**C**) 7 and (**D**) 10 months after injury. (**E**) During the short-term memory evaluation, WT TBI animals spent significantly less time in the target hole than WT sham animals at 7 months postinjury. ** *p* < 0.01 WT TBI vs. S1R-/- TBI ##, ^ *p* < 0.05 WT sham vs. S1R-/- sham, ## *p* < 0.01 WT sham vs. WT TBI, a *p* < 0.05, aa *p* < 0.01 7 vs. 10 months. (**F**) TBI did not induce long-term memory impairments at 7 and 10 months after the injury. WT sham *n* = 9, WT TBI *n* = 10, S1R-/- sham *n* = 7, S1R-/- TBI *n* = 9. ^ *p* < 0.05 WT sham vs. S1R-/- sham, a *p* < 0.05 7 vs. 10 months. (**G**) Heat maps representing weighted occupancy across probe trials conducted 7 and 10 months after TBI. Warmer colors indicate longer dwelling times. The target hole area is denoted with a black circle. (**H**) WT sham and TBI mice showed a time-dependent increase in immobility time after injury. WT sham *n* = 8–9, WT TBI *n* = 6–9, S1R-/- sham *n* = 5–8, S1R-/- TBI *n* = 7–8. * *p* < 0.05 WT TBI vs. S1R-/- TBI, ^ *p* < 0.05, ^^ *p* < 0.01 WT sham vs. S1R-/- sham. All values are presented as means ± standard errors of the means (SEM). *p* values for differences between groups were calculated using repeated measures two-way analysis of variance (RM two-way ANOVA) followed by Fisher’s least-significant difference (LSD) test.

Sensorimotor function was assessed using the neurological severity score (NSS) and rota-rod (RR) test. TBI induced significant neurological impairments in WT TBI mice (*p* = 0.006) but not in S1R-/- TBI mice 24 h after injury compared to the respective sham groups (*p* = 0.191, effect of group (F3,32 = 16.1; *p* < 0.0001), repeated measures (RM) two-way ANOVA followed by Fisher’s least-significant difference (LSD) test, Figure 1A). In addition, S1R-/- TBI mice exhibited a significantly decreased NSS (1.78 ± 0.57) compared to WT TBI mice (4.20 ± 0.79) at 24 h postinjury (*p* = 0.024). Compared to WT sham mice, the NSSs of WT TBI mice indicated sensorimotor deficits for up to 9 months postinjury (*p* < 0.05). From the second week to 9 months postinjury, the NSS was also significantly increased in the S1R-/- TBI group compared with the S1R-/- sham animals. No differences were observed at 12 months postinjury across all experimental groups (*p* > 0.05, Figure 1A).

In the accelerating RR test, WT TBI animals spent less time on the RR 24 h after injury compared to WT sham animals (*p* = 0.038, effect of group (F3,29 = 8, *p* = 0.0006), and time (F4,87 = 5, *p* = 0.002), RM two-way ANOVA followed by Fisher’s LSD test, Figure 1B). We did not observe any impairment of motor coordination in S1R-/- TBI animals compared to S1R-/- sham animals (*p* > 0.05, Figure 1B). Moreover, motor coordination was not significantly different at any time point after injury compared to baseline values in S1R-/- sham and TBI animals (*p* > 0.05). We observed impaired motor coordination in WT sham animals starting at 6 months postinjury compared to baseline measurements (Figure 1B). WT TBI animals spent less time on the RR starting at 24 h after injury compared to baseline values (Figure 1B).

### 2.2. S1R-/- Mice Have Preserved Cognitive Ability after TBI

Spatial learning and memory function were evaluated using the Barnes maze (BM) to test whether any long-term effects on cognitive function were present in WT and S1R-/- mice after TBI. Learning ability was evaluated as the latency to reach the target hole over 4 learning days. Learning was unaffected in all experimental groups at 7 months (RM two-way ANOVA, effect of time F3,87 = 37.75, *p* < 0.0001, effect of group F3,29 = 0.884, *p* = 0.460, Figure 1C) and 10 months after injury (effect of time F3,81 = 3, *p* = 0.020, effect of group F3,27 = 2, *p* = 0.202, Figure 1D). Time spent in the target hole area, which previously contained an escape hole, was measured 24 h (short-term memory) and 7 days (long-term memory) after the last learning day to assess spatial memory processes. TBI induced short-term memory impairments in WT mice (effect of group F3,24 = 7.958, *p* < 0.001, Figure 1E). WT TBI animals spent significantly less time in the target hole than WT sham animals at 7 months postinjury (*p* < 0.01, Figure 1E). At 10 months postinjury, WT TBI animals appeared to perform worse than WT sham animals; however, the difference failed to reach significance (*p* = 0.070, Figure 1E). Short-term memory was not impaired in S1R-/- TBI mice compared to S1R-/- sham mice after injury (*p* > 0.05, Figure 1E). Long-term memory was not affected in any experimental groups after injury (*p* > 0.05, Figure 1F). We also observed that S1R-/- sham and TBI mice displayed improvements in short-term memory function at 10 months compared to 7 months (effect of time F1,8 = 12.3, *p* < 0.01, S1R-/- sham *p* = 0.011, TBI *p* < 0.01, Figure 1E), while WT sham and TBI mice performed similarly over time (*p* > 0.05, Figure 1E).

Next, we used the y-maze test to evaluate spatial working memory. We did not observe any differences in spontaneous alternations between the experimental groups (*p* > 0.05, Table S1), indicating that spatial working memory was not affected after TBI.

### 2.3. S1R-/- Mice Show Reduced Despair-like Behavior and Increased Anxiety, Regardless of Injury Status

Depressive-like behavior was assessed using the tail suspension test (TST). A significant effect of time on immobility in the TST was observed (RM two-way ANOVA, F3,66 = 5.21, *p* = 0.004, Figure 1H). Over the course of the experiment, immobility time was unchanged in S1R-/- sham and TBI mice (*p* > 0.05, Figure 1H). WT sham and TBI mice showed a time-dependent increase in immobility time after injury. WT sham mice spent more time in an immobile state at 10 and 12 months postinjury (*p* = 0.032 and *p* = 0.018, respectively) than at 3 months. WT TBI mice showed a significant increase in immobility at 8 months (*p* = 0.032) postinjury compared to 3 months; however, at later time points, the difference failed to reach significance. In addition, the immobility time was significantly decreased in S1R-/- sham mice compared to WT sham mice at 3 (*p* = 0.047), 8 (*p* = 0.045) and 12 months postinjury (*p* < 0.01, Figure 1H). S1R-/- TBI mice spent less time in an immobile state than WT TBI mice at 8 months postinjury (*p* = 0.010, Figure 1H).

The evaluation of anxiety-like behavior by quantification of the cumulative time spent in the closed and open quadrants showed a significant group effect (RM two-way ANOVA, F3,34 = 4.98, *p* = 0.005, Table S2). Post hoc tests revealed that the WT sham and TBI groups spent equivalent amounts of time in the open quadrants of the maze, indicating no change in anxiety as a consequence of TBI (*p* > 0.05, Table S2). We observed differences between WT and S1R-/- mice, regardless of their injury status. S1R-/- sham mice spent less time in open quadrants than WT sham mice 1 month after the injury (*p* = 0.025, Table S2). By 8 months, S1R-/- sham mice tended to spend less time in open quadrants than WT sham mice (*p* = 0.058). Similarly, S1R-/- TBI mice spent less time in open quadrants than WT TBI mice at 1 (*p* = 0.081) and 8 months (*p* = 0.014) postinjury.

No significant differences were observed between experimental groups in the distance traveled, suggesting that all groups retained similar motor activity and a similar ability to explore the open field arena within a 12-month period after injury (*p* > 0.05, Table S3). In addition, total activity did not differ between experimental groups in metabolic cages with running wheels (*p* > 0.05, Table S3). No significant changes were observed in drinking and feeding behaviors at 1, 3, and 6 months after TBI (*p* > 0.05, Table S4).

### 2.4. S1R-/- Mice Exhibit Decreased Glial Fibrillary Acidic Protein (GFAP) Staining in the Molecular Layer of the Cerebellum

**Figure 2:**
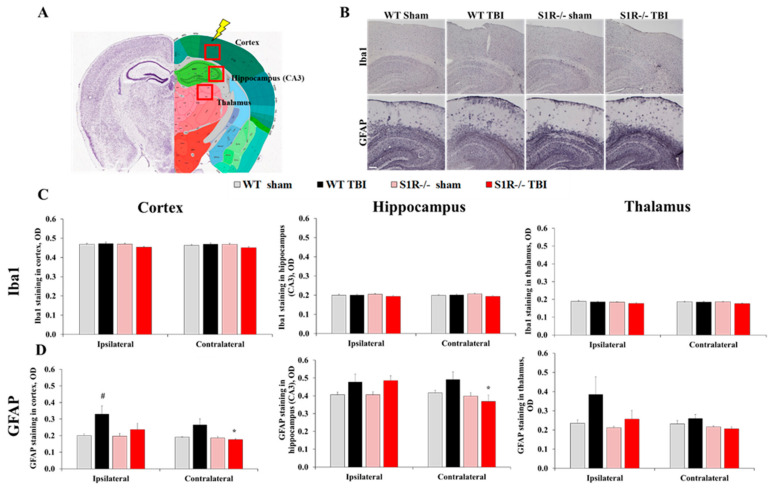
Staining of microglia (Iba1) and astrocytes (GFAP) in WT and S1R-/- mouse brain slices at 12 months postinjury. (**A**) The anatomical positions of the analyzed brain regions were validated using the Allen Mouse Brain Atlas (http://mouse.brain-map.org/static/atlas, accessed on 15 October 2021). (**B**) Representative images of Iba1 and GFAP staining in the cortex and hippocampus (total magnification = 40×, scale bar = 100 µm). Measured optical densities (OD) of the staining intensity of (**C**) Iba1 and (**D**) GFAP in the cortex, hippocampus, and thalamus between the WT and S1R-/- animal groups. All values are presented as the means ± SEM. *p* values for differences between groups were calculated using the Mann–Whitney U-test, (*n* = 5): * *p* < 0.05 WT vs. S1R-/-, # *p* < 0.05 WT sham vs. WT TBI.

**Figure 3:**
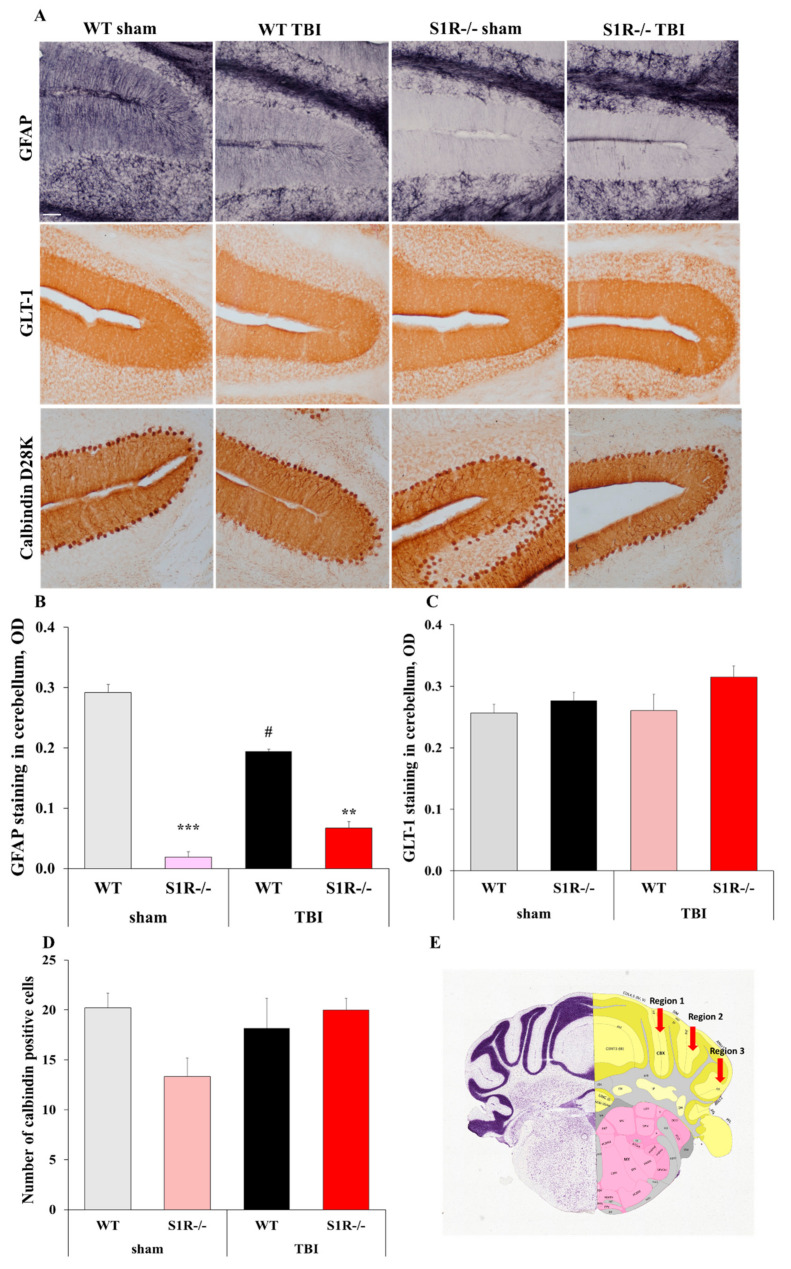
Staining of astrocytes (GFAP, Glt-1) and Purkinje cells (Calbindin D28K) in WT and S1R-/- mouse cerebellum slices at 12 months postinjury. (**A**) Representative images of GFAP, Glt-1, and Calbindin D28K staining in the grey matter of the cerebellum (total magnification = 100×, scale bar = 50 µm). OD of the GFAP (**B**), Glt-1 (**C**) staining intensity in the molecular layer of the cerebellum between the WT and S1R-/- experimental groups. (**D**) Total cell counts of Purkinje cells in the molecular layer of the cerebellum between WT and S1R-/- experimental groups. (**E**) Anatomical positions of the analyzed regions in the cerebellum (red arrows). All values are presented as the means ±SEM. *p* values for differences between groups were calculated using the Mann–Whitney U-test, (*n* = 5): ** *p <* 0.01, *** *p* < 0.001 WT vs. S1R-/-, # *p* < 0.05 WT sham vs. WT TBI.

We compared the Iba1 and GFAP staining intensity between the experimental groups of WT and S1R-/- mice to evaluate microglial (Iba1) and astrocyte (GFAP) activation 12 months after TBI (Figure 2B). We examined the cortex, hippocampus (hippocampal cornu ammonis 3 (CA3) region), and thalamus (lateral nucleus of thalamus) as respective impact site regions. We also evaluated the astrocyte (GFAP, Glt-1) staining intensity and number of Purkinje cells (Calbindin D28K) in the grey matter of the cerebellum, which is associated with motor-related and cognitive functions that were prominently affected after TBI. TBI induced a significant increase in GFAP staining in the ipsilateral and contralateral cortex of WT mice compared to sham mice (Mann–Whitney U-test, *p* = 0.015 and *p* = 0.015, respectively, Figure 2D). S1R-/- TBI mice showed significantly decreased GFAP staining in the contralateral cortex compared with WT TBI mice (Mann–Whitney U-test, *p* = 0.007, Figure 2D). TBI induced a significant decrease in GFAP staining in the molecular layer of the cerebellum in WT mice compared to sham mice (Mann–Whitney U-test, *p* = 0.037, Figure 3B). Surprisingly, S1R-/- animals displayed almost no GFAP staining in the molecular layer of the cerebellum after injury (Figure 3B). GFAP staining in the cerebellum of S1R-/- mice was significantly different from that in WT animals (*p* < 0.00 S1R-/- sham vs. WT sham, *p* = 0.019 S1R-/- TBI vs. WT TBI, Figure 3B). The intensity of GFAP staining was also significantly decreased at baseline (before TBI) in S1R-/- mice compared to WT mice (Mann–Whitney U-test, *p* < 0.001, Figure S1). There were no significant differences in Glt-1 staining intensity and number of Purkinje cells in the grey matter of the cerebellum between experimental groups (Mann–Whitney U-test, *p* > 0.05, Figure 3C,D). No differences in the Iba1 staining intensity in the cortex, hippocampus, or thalamus were observed between WT and S1R-/- mice from either the sham or TBI groups (Figure 2C).

### 2.5. Health Outcome Measures after TBI

S1R deficiency did not affect the general health condition of mice after TBI. Postinjury apnea was not significantly different between the WT TBI (46.7 ± 13.3) and S1R-/- TBI groups (28.3 ± 6.2) (*p* = 0.228, Figure S2). Overall mortality was not significantly affected by the injury (chi-square test, *p* = 0.175, Figure S2). After injury, the body weight was not significantly different between experimental groups, indicating that mice maintained good general health throughout the study (Figure S2). Posttraumatic seizure episodes were observed in both WT (5/12) and S1R-/- (1/12) animals. The first episode of seizures in each group was observed at 8 days postinjury. None of the sham animals displayed seizures after the injury.

### 2.6. BD-1063 Does Not Influence Acute Injury Measures or Behavioral and Histological Outcomes Following TBI

**Figure 4:**
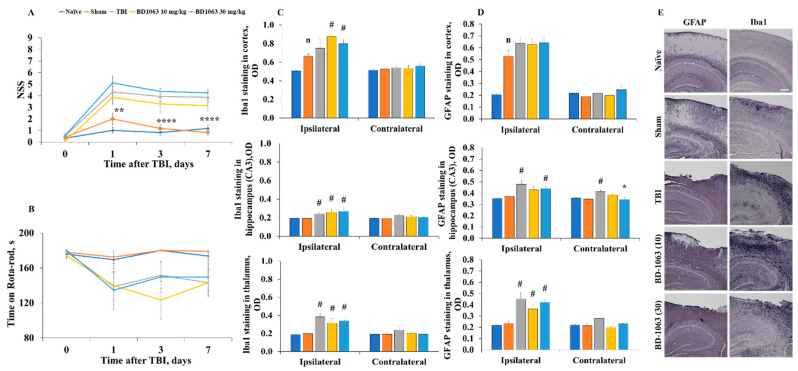
Posttraumatic behavioral and histological outcomes after pretreatment with the S1R antagonist BD-1063. (**A**) TBI mice had a significantly higher NSS up to 7 days postinjury. (**B**) All experimental groups spent similar time on the RR. BD-1063 treatment did not influence behavioral outcomes after TBI. Data are presented as the means ± SEM (naïve *n* = 6, sham *n* = 9, TBI *n* = 15, BD-1063 10 mg/kg *n* = 7, BD-1063 30 mg/kg *n* = 8). *p* values for differences between groups were calculated using RM two-way ANOVA followed by Fisher’s LSD test: * *p* < 0.05, ** *p* < 0.01, **** *p* < 0.0001 sham vs. TB. OD of the intensity of (**C**) Iba1 and (**D**) GFAP staining in the cortex, hippocampus, and thalamus. TBI induced significantly increased staining for Iba1 in the ipsilateral thalamus and for GFAP in the ipsilateral hippocampus and thalamus. (**E**) Representative images of Iba1 and GFAP staining at 7 days postinjury (total magnification = 40×, scale bar = 100 µm). Data are presented as the means ± SEM (*n* = 5). *p* values for differences between groups were calculated using the Kruskal–Wallis test followed by Dunn’s test: # *p* < 0.05 compared with the sham group, * *p* < 0.05 compared with the TBI group. Differences between naïve and sham groups were calculated using the Mann–Whitney U-test: *^n^ p* < 0.05 compared with the naïve animals.

An assessment of NSS from baseline to 7 days after TBI revealed significant time (F2,48 = 5, *p* = 0.017) and group (F3,33 = 8, *p* = 0.0003) effects (RM two-way ANOVA, followed by Fisher’s LSD test, Figure 4A). The NSS was significantly higher in TBI animals than in sham animals at 1, 3, and 7 days postinjury. In the RR test, the TBI and TBI + BD1063 10 and 30 mg/kg groups spent similar amounts of time on the RR as the sham animals (effect of time F2,49 = 0.2, *p* = 0.78 and group F3,33 = 1, *p* = 0.35, RM two-way ANOVA, Fisher’s LSD test, Figure 4B). BD-1063 treatment did not affect sensorimotor ability and motor coordination. After the subcutaneous BD-1063 injection at a dose of 30 mg/kg for 7 days, the detected concentration of the test compound in the brain was 6.3 ± 0.03 µg/g. However, in the brain tissue, no significant differences were observed in S1R gene expression between the BD-1063 30 mg/kg treatment (1.1-fold) and saline (1.0-fold) groups.

We performed immunohistochemical staining to assess TBI-induced activation of microglia (Iba1) and astrocytes (GFAP) in the experimental groups (Figure 4C). The TBI group displayed significant increases in the intensity of Iba1 staining in the ipsilateral thalamus (lateral nucleus of the thalamus, *p* = 0.023, Kruskal–Wallis test followed by Dunn’s test; Figure 4C) and GFAP staining in the ipsilateral hippocampus (hippocampal cornu ammonis 3 (CA3) region, *p* = 0.018, Kruskal–Wallis test followed by Dunn’s test; Figure 4D) and thalamus (*p* = 0.007, Kruskal–Wallis test followed by Dunn’s test; Figure 4D). Increased GFAP staining was also observed in the contralateral hippocampus of the TBI group (*p* = 0.0043, Kruskal–Wallis test followed by Dunn’s test; Figure 4C). Due to the surgical procedure, sham animals presented significantly increased Iba1 (Figure 4C) and GFAP (Figure 4D) staining in the ipsilateral cortex 7 days after TBI (*p* = 0.015 compared with naïve animals, Mann–Whitney U-test), while no increase in staining was observed in the hippocampus and thalamus (*p* > 0.05, Figure 4C,D). Treatment with BD-1063 did not result in any significant differences in Iba1 and GFAP staining in the cortex, hippocampus, or thalamus compared with the TBI group (Figure 4C,D).

The duration of apnea and time to regain the righting reflex were equal for the TBI and BD-1063 treatment groups (ordinary one-way ANOVA, *p* > 0.05). No differences in body weight were observed between experimental groups after injury (RM two-way ANOVA, *p* > 0.05).

## 3. Discussion

In the present study, we used a lateral fluid percussion injury model of TBI to evaluate the role of S1R in the development of neurological deficits for up to 12 months after TBI. This model resulted in neurological and motor dysfunction in WT mice that was less pronounced in S1R-/- mice at 24 h after injury. TBI induced long-term cognitive impairments in WT mice but not in S1R-/- mice. WT TBI animals displayed significantly higher astroglia-related GFAP protein expression levels in the cortex, while GFAP protein expression levels in both S1R-/- sham and S1R-/- TBI animals were similar to those in the WT sham group at 12 months postinjury. S1R-/- animals exhibited substantially reduced GFAP expression in Bergmann glial cells in the molecular layer of the cerebellum compared to WT mice before and 12 months after TBI. In addition, we observed age-related behavioral changes in WT mice but not in S1R-/- mice. S1R-/- mice displayed preserved motor coordination and reduced despair-like behavior compared to WT mice over a 12-month period.

TBI is a chronic health condition with serious long-term consequences such as motor dysfunction, depression, cognitive deficits, and emotional changes [4,8,19]. Currently, no effective treatment is available for TBI-induced deficits other than supportive therapy [20]. Therefore, research on treatments that prevent the progression of brain damage after TBI is necessary. Drugs interacting with S1R have potential as treatments for neurological diseases, including TBI [13,14,21]. Here, S1R deficiency was associated with improved motor function and diminished neurological deficits in the acute phase after TBI. Previous studies have also shown that S1R deficiency attenuates neurodegenerative processes. For example, in the MPTP-induced Parkinsonism model, motor deficits and dopaminergic neuron death were less pronounced in S1R-/- mice than in WT mice [22]. Another study indicated that S1R-/- mice present reduced mechanical allodynia, macrophage/monocyte infiltration, and levels of the chemokine CCL2 in dorsal root ganglia after spinal nerve injury [23]. S1R deficiency alters cognitive function, especially in older mice [24], and is associated with more pronounced learning deficits and toxicity in APPSwe AD mice [25]. In contrast, we showed no changes in the spatial learning and memory abilities of S1R-/- mice for up to 10 months after TBI. In addition, we observed that both sham and TBI S1R-/- mice displayed less despair behavior than WT mice over a 12-month period. Our findings indicate an important role for S1R in the development of TBI-induced neurobehavioral deficits in the acute and chronic phases after TBI.

In recent years, accumulating evidence has shown that S1R is implicated in the modulation of neuronal physiology and synaptic plasticity [26]. Various anti-inflammatory mechanisms have been postulated for S1R ligands that might account for their potential neuroprotective effects [27]. Astrocytes and microglia are considered key players in the initiation of an inflammatory response after injury [28]. Astrocytosis occurs in concert with neuronal degeneration [29,30] and has been noted in rodents, primarily in the cortex, within the first 24 h [31] and up to 12 months after TBI [32]. In the present study, TBI-induced astrocyte activation in the brain tissue was reduced in S1R-/- mice, suggesting that S1R deficiency prevents development of neurodegenerative processes in brain tissue 12 months postinjury. A recent study found that S1R deficiency reduced MPTP-induced astrocyte activation in the substantia nigra [22]. Another study documented increased GFAP expression in mixed astrocyte–neuronal cultures derived from S1R-/- mice, which seemed to counterbalance the cellular response to stressful conditions. [33] Similar to astrocytosis, in the context of many neurological disorders, chronic microglial activation is responsible for neurodegeneration [28]. Recent studies have shown that S1R agonists acutely decrease microglial activation following brain injury in vivo [13,14,15,21]. Although microglia remain highly activated in different brain regions up to 1 year after controlled cortical impact injury (CCI) [34], we did not observe any differences between the sham and TBI groups in terms of microglial activation at 12 months postinjury. The discrepancy in microglial activation might be due to the use of different types of TBI animal models. The CCI model produces more extensive damage to brain tissue [34] than the latFPI model used in the present study.

Impairments in motor function and coordination are common consequences of TBI and are usually associated with injury to the sensorimotor cortex [35]. However, the cerebellum also plays an important role in the control and coordination of movement that may be specifically related to motor dysfunction after TBI [36]. Here, we showed that both sham and TBI WT mice developed impairments in motor coordination with age, while S1R-/- mice exhibited preserved motor function. One of the possible explanations for motor dysfunction in WT mice is increased astrocyte activation in the cerebellum. Cerebellar astrocytosis reduces the survival of Purkinje cells, which are associated with movement and coordination, and leads to cerebellar dysfunction and motor impairments after TBI [37,38,39]. Here, S1R-/- mice displayed preserved motor coordination and almost no GFAP expression in the molecular layer of the cerebellum after TBI. Similarly, GFAP expression was substantially decreased in S1R-/- sham mice compared to WT mice. Based on localization, GFAP-positive astrocytes are Bergmann glia [40]. Bergmann glia directly regulate Purkinje cells and influence motor behavior [41,42]. We found no significant difference in total number of PCs in S1R-/- mice. This is in line with previous findings in which PC degeneration and motor dysfunction was associated with both increased GFAP expression and ablation of Bergmann glia [43,44,45]. Our data show that the improved motor coordination exhibited by S1R-/- mice could be due to decreased GFAP expression in Bergmann glial cells in the molecular layer of cerebellum. These results suggest important involvement of S1R in the regulation of information processing in the cerebellum and control mechanisms of motor behavior. Therefore, decreasing GFAP expression in Bergmann glia may represent a novel therapeutic strategy for motor dysfunction during ageing.

Since S1R-/- mice showed acute improvements in neurological function after TBI, we examined whether pharmacological treatment with an S1R antagonist influenced behavioral and histological outcomes after injury. Treatment with BD-1063 did not affect TBI-induced neurological deficits. Although we measured a sufficient concentration of BD-1063 in the brain tissue (6.3 ± 0.03 µg/g), no significant difference in S1R gene expression was observed between saline- and BD-1063-treated animals. S1R agonists are able to attenuate neurological deficits and lessen TBI-induced neurodegeneration by reducing microglial activation following brain injury in vivo [13,14,21]. In previous studies, BD-1063 was used to block the effect of S1R agonists [46,47]. Recently, oral treatment with BD-1063 induced neuroprotection in an SCI model by increasing the number of surviving motor neurons and decreasing microglial activation in the ventral horns of L4-L5 spinal segments [48]. Thus, different S1R ligands may act differently and even adversely in neuroprotection.

In conclusion, S1R deficiency led to improved neurological and motor coordination, reduced despair-like behavior, preserved long-term cognitive function, and prevented TBI-induced neuroinflammation 12 months postinjury. S1R deficiency was associated with reduced GFAP expression in Bergmann glial cells in the cerebellum. These findings suggest a role for S1R in the pathogenesis of TBI and cerebellum-mediated motor behavior.

## 4. Materials and Methods

### 4.1. Animals and Experimental Design

Twenty-one S1R knockout (S1R-/-) male mice aged 10 weeks (Laboratorios Dr. Esteve S.A., Barcelona, Spain) were used to explore the effects of genetic deletion of S1R on traumatic brain injury. S1R-/- mice on a CD-1 genetic background were generated as described previously [49]. Twenty-two wild-type male mice (WT) aged 10 weeks (HSD:ICR(CD-1^®^), ENVIGO, Venray, Netherlands) of the same genetic background as the S1R-/- mice were used. Sixty-nine 12- to 14-week-old WT male mice were used to evaluate the effects of the S1R antagonist BD-1063 on injury. Ten 36-week-old male Swiss-Webster mice (Laboratory Animal Centre, University of Tartu, Tartu, Estonia) were used to investigate BD-1063 concentrations and S1R expression in brain tissue. Three S1R-/- mice (Laboratorios Dr. Esteve S.A., Barcelona, Spain) and three WT males aged 10 weeks were used to evaluate astrocyte activation in the cerebellum of intact animals. All animals were housed under standard conditions (21–23 °C, 12 h dark–light cycle, with lights off at 8 a.m. and on at 8 p.m.) with unlimited access to standard food (Lactamin AB, Mjölby, Sweden) and water in an individually ventilated cage housing system (Allentown Inc., Allentown, NJ, USA). Each cage contained EcoPure™ wood chip shavings (Datesand, Cheshire, UK), nesting material, and wood blocks from TAPVEI (TAPVEI, Paekna, Estonia). For enrichment, a transparent tinted (red) nontoxic durable polycarbonate safe harbor mouse retreat (Animalab, Poznan, Poland) was used. The mice were housed in groups of up to five mice per standard cage (38 × 19 × 13 cm). All studies involving animals were reported in accordance with the ARRIVE guidelines [50,51]. The experimental procedures were performed in accordance with the guidelines reported in EU Directive 2010/63/EU and with local laws and policies; all of the procedures were approved by the Latvian Animal Protection Ethical Committee of Food and Veterinary Service in Riga, Latvia.

**Figure 5:**
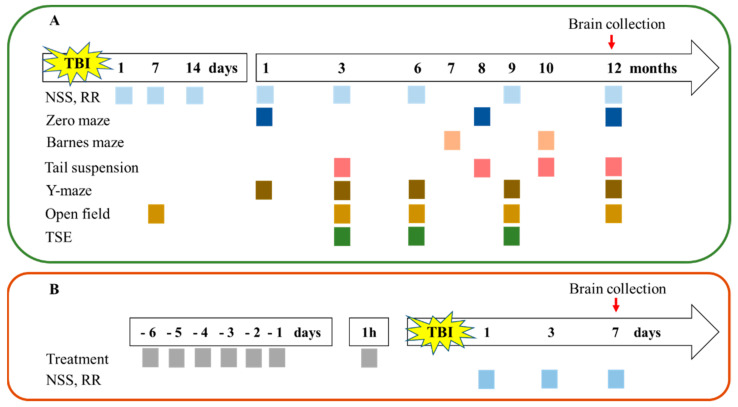
Schematic depicting the experimental design. (**A**) S1R-/- and WT mice were subjected to latFPI, and behavioral outcomes were assessed for up to 12 months postinjury. (**B**) BD-1063 was administered to evaluate the effects of pharmacological blockade of S1R. Behavioral outcomes were evaluated for up to 7 days postinjury. Brain tissues were collected from animals in both experiments for histological analysis at the end point. TBI—traumatic brain injury, NSS—neurological severity score, RR—rota-rod, TSE—PhenoMaster behavioral phenotyping.

A subset of animals subjected to experimental lateral fluid percussion injury was randomly separated into four experimental groups: WT sham (*n* = 10), S1R-/- sham (*n* = 10), WT TBI (*n* = 12), and S1R-/- TBI (*n* = 12). Mice were weighed before injury (baseline measurement), on the first day after the injury, and then weekly throughout the study as a measure of general health. Body weight was reported as a percent change relative to baseline. Behavioral tests were performed at baseline and 1, 7, and 14 days and 1, 3, 6, 9, and 12 months after injury. Mice were euthanized at 12 months postinjury, and brain tissue was collected for histological analysis. The study design is presented in Figure 5A.

One WT mouse and two S1R-/- animals were excluded due to dural breach during the surgical procedure. No animal subjected to sham injury was excluded. Over the 12 months of this study, 10 mice died prematurely (WT TBI *n* = 3, S1R-/- TBI *n* = 4, S1R-/- sham *n* = 3, Figure S2). For behavioral tests, all data points (including those generated by mice that eventually died prematurely) were included. For the histological analysis, only mice that survived up to the 12-month time point were included, and all available brain tissues were analyzed.

Treatment with the S1R antagonist BD-1063 was performed to evaluate the effect of pharmacological blockade of S1R. Mice were randomly divided into five groups: naïve (*n* = 6), sham (*n* = 12), TBI (*n* = 18), TBI + BD-1063 10 mg/kg (*n* = 16), and TBI + BD-1063 30 mg/kg (*n* = 16). The dose of BD-1063 was chosen based on previous in vivo studies [47]. Naïve and sham groups received the corresponding vehicle with the same volume and treatment regimen. BD-1063 (TOCRIS, Bristol, UK) at doses of 10 or 30 mg/kg or vehicle (0.9% NaCl solution) was injected subcutaneously (sc) for 6 consecutive days prior to TBI. The last injection (total of 7 injections) was administered 1 h before TBI. Mice were weighed before (baseline measurement) and 1, 2, 3, and 7 days after injury as a measure of general health. Body weight was reported as a percent change relative to baseline. Behavioral tests were performed at baseline and 1, 3, and 7 days after injury. The study design is presented in Figure 5B.

Two mice (sham *n* = 1, TBI *n* = 1) died spontaneously during the pretreatment period. Two mice died during surgery (TBI *n* = 1, TBI + BD-1063 30 mg/kg *n* = 1). Fifteen animals were excluded due to dural breach during the surgical procedure (sham *n* = 2, TBI *n* = 1, TBI + BD-1063 10 mg/kg *n* = 7, TBI + BD-1063 30 mg/kg *n* = 5). Four mice died after TBI (TBI + BD-1063 10 mg/kg *n* = 2, TBI + BD-1063 30 mg/kg *n* = 2). For behavioral analyses, all data points (including those generated by mice that died after TBI) were included. Mice were euthanized at 7 days postinjury, and brain tissue was collected for histological analysis. Solutions for BD-1063 injections were prepared immediately before use in sterile saline from stock solutions with a concentration of 10 mg/mL that were previously prepared and stored at −20 °C. All analyses were conducted by investigators blinded to the group allocation. Behavioral tests were performed during the dark phase (from 9 a.m. to 4 p.m.).

### 4.2. Lateral Fluid Percussion Injury

The lateral fluid percussion injury (latFPI) model was established as previously described [52]. Briefly, anesthesia was induced with 4% isoflurane (Chemical Point, Deisenhofen, Germany) contained in a mixture of oxygen and nitrous oxide (70:30, AGA, Riga, Latvia) and maintained with 2% isoflurane using a face mask. Before trauma induction, the mice received a sc injection of tramadol (KRKA, Novo Mesto, Slovenia) (10 mg/kg). A craniectomy was performed using a 3 mm (outside diameter) circular trephine over the parietal region, 2 mm posterior to bregma and 2 mm right of midline. Injury was induced using a fluid percussion device connected to a pressure measurement instrument (Model FP 302, AmScience Instruments, Richmond, VA, USA). The duration of apnea was monitored immediately after the injury. Sham animals underwent the same procedures as the animals in the latFPI group except for the induction of trauma. Mice with weight loss >20%, dural breech, or mortality within 24 h postinjury were excluded from the study.

### 4.3. Neurological Severity Score

The neurobehavioral status of the mice was assessed using the NSS as previously described [53]. The NSS consisted of nine individual clinical parameters, including tasks on motor function, alertness, and physiological behavior. The mice were assessed for the following items: presence of paresis; impairment of seeking behavior; absence of perceptible startle reflex; inability to get down a rectangle platform (34 × 27 cm); inability to walk on 3-, 2-, and 1-cm wide beams; and inability to balance on a vertical beam of 7 mm width and horizontal round stick of 5 mm diameter for at least 15 s. If a mouse showed impairment on one of these items, a value of 1 was added to its NSS score. Higher scores on the NSS thus indicate greater neurological impairment.

### 4.4. Rota-Rod Test

A rota-rod (Model 47600; Ugo Basile) test with slight modification was used to measure motor coordination [54]. Briefly, mice were pretrained on the rota-rod apparatus (5 rpm) with 2 sessions per animal, each lasting for 240 s. On the experimental day, mice were placed on the rod with an accelerating rotating speed from 5 to 25 rpm over a period of 240 s with a 30 min rest between trials. Time spent walking on the accelerating rota-rod before falling off was measured. The mean of two trials was calculated for each mouse.

### 4.5. Y-Maze Test

Working memory performance was assessed by recording spontaneous alternation behavior in a Y-maze, as previously described [55]. The mice were individually placed at the end of one arm in a symmetrical Y-shaped runway (arm length 35 cm, width 5 cm, height 21 cm) and allowed to explore the maze for 5 min. A spontaneous alternation behavior was defined as the entry into all three arms on consecutive choices in overlapping triplet sets (i.e., ABC, BCA, CBA). The percent spontaneous alternation behavior was calculated as the ratio of actual to possible alternations (defined as the total number of arm entries − 2) × 100.

### 4.6. Tail Suspension Test

Despair-like behavior was assessed by tail suspension test, as previously described [56]. Briefly, each animal was suspended with tape (17 cm) from a horizontal rod elevated 30 cm above a clean cage for 6 min. To prevent mice from climbing their tails, a clear hollow cylinder (Ø = 4.5 cm, h = 5.5 cm) was placed around the tail before suspension. Mice were recorded for 6 min using the digital HD video camera recorder (Handycam HDR-CX11E, Sony Corporation, Tokyo, Japan) and immobilization was analyzed during the last 4 min. Immobility included motionless time as well as passive swinging caused by momentum from movement.

### 4.7. Open Field Test

Locomotor and anxiety-like behavior was assessed using the open-field test. The open field was a square arena (44 × 44 cm) shielded by 30 cm high opaque walls. Square area (20 × 20 cm) was defined as the center. The mouse was gently placed in the center of the field and allowed to explore for 12 min. The distance traveled, velocity, and time spent in the center were recorded and analyzed for 4 min periods using an EthoVision video tracking system (version XT 11.5, Noldus Information Technology, Wageningen, The Netherlands).

### 4.8. Elevated Zero Maze Test

Anxiety was assessed using the elevated zero maze test. The apparatus consisted of a black circular platform (width: 4.5 cm, diameter 50 cm), placed 44 cm from the ground and divided into four equal quadrants. Two opposite quadrants had 15 cm high dark, opaque walls (closed quadrants), while the other two had no walls (open quadrants). Mice were placed in the center of open quadrant, and allowed to explore for 5 min. Time spent in each quadrant was registered using an EthoVision video tracking system (version XT 11.5; Noldus, Wageningen, The Netherlands).

### 4.9. Barnes Maze Test

Spatial learning and memory were assessed using the Barnes maze test as previously described with slight modifications [57]. The test was performed on a brightly lit grey circular platform (diameter 92 cm) with 20 equally spaced holes (diameter 5 cm) located around the perimeter, an escape box fitted under in one of the holes, and visual cues in the periphery. On the first day, the animal was placed at the center of the platform using a glass beaker and allowed to acclimate for 2 min; the mouse was then guided to the target hole and allowed to stay there for 1 min. Mice then underwent four days of learning consisting of three consecutive trials separated by brief returns to their home cages. If the mouse did not enter the escape box (defined as all four paws leaving the surface of the platform) within the 180 s trial, the experimenter guided the mouse to the escape hole as before and allowed it to rest for 1 min before returning the animal to its home cage. During the 90 s probe trials (24 h and 7 days after the last learning), the escape box was removed, and the time spent in the area of the target hole was recorded using the EthoVision (version XT 11.5; Noldus, Wageningen, The Netherlands).

### 4.10. TSE PhenoMaster System (Indirect Calorimetry)

The feeding and drinking behavior and locomotor activity were tested using an eight-cage calorimetry system (PhenoMaster, TSE Systems, Bad Homburg, Germany), which allowed continuous and undisturbed recording. Mouse behaviors were continuously recorded for 72 h with the following measurements recorded every 15 min: water intake (mg/kg body weight), food intake (g/kg body weight), and locomotor activity (voluntary running wheel).

### 4.11. Determination of BD-1063 in the Brain Tissue Using UPLC⁄MS

The concentration of BD-1063 in brain tissue was measured using ultra-performance liquid chromatography–tandem mass spectrometry (UPLC⁄MS). BD-1063 was administered in mice for 7 consecutive days prior to tissue collection. Animals were euthanized 1 h after the last sc administration of BD-1063 at a dose of 30 mg/kg. Brain tissues were collected immediately after the decapitation of animals. The brains were divided into two hemispheres and one of the hemispheres was used for BD-1063 measurements, while the second half was used for quantitative PCR analysis for S1R. The brain hemisphere was homogenized in ice-cold Milli-Q water at a *w*/*v* ratio of 1:5 using bead-beating technology (Omni Bead ruptor 24; Omni International, Kennesaw, GA, USA) in 2 mL tubes [58]. The obtained homogenate was centrifuged at 16,500 rpm for 10 min at 4 °C. The supernatant was then decanted, and the pellet was homogenized in the same volume of Milli-Q water as previously used. The obtained homogenate was centrifuged at 16,500 rpm for 10 min at 4 °C. The supernatants were combined and stored at −80 °C until use.

Brain tissue extract (100 μL) was mixed with 400 μL of IS (Verapamil) solution in acetonitrile to precipitate proteins. The tubes were vortexed to mix and centrifuged at 10,000 rpm for 10 min. A volume of 400 μL of supernatant was transferred to chromatographic vial, diluted with 400 μL of 0.1% formic acid, and subjected to UPLC/MS analysis. The calibration curve was constructed by plotting ratios of peak areas (analyte/IS) versus BD-1063 concentration over the range of 4 to 1000 ng/mL.

UPLC was carried out using the Waters Acquity UPLC system equipped with the Acquity BEH C18 column (2.1 × 50mm, 1.7 μm). The mobile phase consisted of A (aqueous 0.1% formic acid) and B (acetonitrile) at a flow rate of 0.4 mL/min, using a gradient elution: initial—5% B, 2.5 min—50% B, 3.5 min—98% B, 4.5min—98% B, 4.7 min—5% B, 6 min—5% B. The injection volume was 10 μL. The column temperature was 30 °C.

MS analysis was performed on a triple quadrupole mass spectrometer Quattro micro-Waters in ESI positive mode using multiple reaction monitoring of two transitions for BD-1063 from *m*/*z* 273.1 to *m*/*z* 137.1 and from *m*/*z* 273.1 to *m*/*z* 173.1 and the transition for verapamil from *m*/*z* 455.3 to *m*/*z* 165.2 (cone voltage, 30 V; collision energy, 25 eV). The MS conditions were as follows: desolvation gas (N2) flow, 800 L/h; desolvation temperature, 400 °C; ESI source temperature, 120 °C; and capillary voltage, 3.0 kV. Quantitative analysis was achieved using QuanLynx4.1 software (Waters).

### 4.12. Quantitative PCR

Brain hemispheres were stored at −80 °C until isolation of RNA. Total RNA was isolated, first-strand cDNA was synthesized, and quantitative PCR analysis for Sig1R was performed as described previously [59]. The primer sequences used in this study were as follows: Sigmar1 (NM_011014.3), 5′-CAT TCG GGA CGA TAC TGG-3′ (forward) and 5′-CCT GGG TAG AAG ACC TCA CTT TT-3′ (reverse) and Actb (NM_007393.5), 5′-CCT CTA TGC CAA CAC AGT GC-3′ (forward) and 5′-CAT CGT ACT CCT GCT TGC TG-3′ (reverse). The primers were obtained from Metabion, Germany. The relative expression levels for each gene were calculated using the ΔΔCt method, normalized to the expression of β-actin, and compared to the expression levels of control group animals.

### 4.13. Immunohistochemistry

Mice were anesthetized using intraperitoneal administration of ketamine (200 mg/kg) and xylazine (15 mg/kg). The depth of anesthesia was monitored by toe pinch. Animals were transcardially perfused at a speed of 3 mL/min with 0.01 M phosphate-buffered saline (PBS, pH = 7.4) for 5 min until the blood was completely removed from the tissue. After perfusion, brains were carefully dissected and fixed in 4% PFA overnight at 4 °C. Brains were cryoprotected with a 10–20–30% sucrose gradient over 72 h. Coronal sections (35 μm) were cut using a Leica CM1850 cryostat (Leica Biosystems, Buffalo Grove, IL, USA).

Samples were embedded in an optimal cutting temperature compound (Tissue-Tek^®^ O.C.T.™ Compound, Sakura Finetek Europe B.V., Alphen aan den Rijn, The Netherlands) and placed in a dry ice/isopropanol slurry. Frozen samples were stored at −80 °C. Coronal sections (35 μm thick) of brain were cut using a cryostat Leica CM1850 (Leica Biosystems, Buffalo Grove, IL, USA) and stored in antifreeze buffer at −20 °C until staining was performed. The immunohistochemistry of free-floating sections was determined based on a method described previously [59]. The following primary antibodies were used in this study: rabbit anti-Iba1 antibody (1:2000; Abcam, Cat# ab153696, Cambridge, UK), rabbit anti-GFAP antibody (1:2000, Abcam, Cat# ab7260, Cambridge, UK), mouse anti-Calbindin D28K antibody (1:500, Santa Cruz Biotechnology, Cat# sc365360, Dallas, TX, USA), rabbit anti-EAAT2 antibody (1:5000, Abcam, Cat# ab205248, Cambridge, UK). The goat anti-rabbit IgG (H + L) (1:1000, Invitrogen, Cat# 65-6140, Carlsbad, CA, USA) and goat anti-mouse IgG (H + L) (1:1000, Invitrogen, Cat# 31800, Carlsbad, CA, USA) biotinylated antibodies were used as secondary antibodies. Samples stained with biotinylated antibody and incubated with streptavidin (HRP) (1:1000, Abcam, Cat# ab7403, Cambridge, UK) were processed with freshly prepared DAB reagent.

Images were taken with a Nikon Eclipse TE300 microscope (Nikon Instruments, Tokyo, Japan). Identical brain sections corresponding to identical anatomical structures were used for the analysis. The structures were validated using Allen Mouse Brain atlas (http://mouse.brain-map.org/static/atlas, accessed on 15 October 2021). For each antibody staining experiment, sections from all animals were processed in the same staining tray. Staining was quantified using ImageJ software (ImageJ v1.52a). Negative controls replacing the primary antibody with a buffer solution (TBS-T) only were performed. Eight-bit images were generated from the pictures and were cropped to contain the regions of interest. Means of optical density (OD) were used to quantify the staining intensity of Iba1 and GFAP in specific brain structures. For OD analysis, calibration was performed in accordance with the instructions on the ImageJ software website (https://imagej.nih.gov/ij/docs/examples/calibration/, accessed on 15 October 2021). The number of Purkinje cells (PC) in the cerebellum was assessed in 10× magnification pictures. For each animal, six pictures of different areas of the cerebellum were taken, all including the PC layer. The number of PCs in each picture was counted and corrected for the length of the PC layer, which was measured by drawing a line through the PC layer using ImageJ and measuring the length of the line. Two criteria were used for cell counts: the cell body (1) was well defined (i.e., not blurred), and (2) had the general characteristic morphology of a PC (i.e., round cell body approximately 25–30 μm diameter located between the granule cell and molecular layers).

### 4.14. Statistical Analysis

The statistical analysis and visualization were performed using GraphPad Prism software (GraphPad Prism Software, Inc., La Jolla, CA, USA). The data were found to be normally distributed using the Shapiro–Wilk test. For behavioral tests, two-way mixed design repeated measures analysis of variance was used to calculate group differences at each time point. In all comparisons, Fisher’s least-significant difference post hoc analysis was used when appropriate (one or both main factors were statistically significant). For histological evaluations, the Mann–Whitney U-test or Kruskal–Wallis test with Dunn’s post hoc test was used. For overall mortality, the chi-square test was used for comparisons between groups. All data are presented as the means ± standard errors of the means. *p* values less than 0.05 were considered significant.

## Data Availability

Data are available from the authors under reasonable request.

## Supplementary Materials

The following are available online at https://www.mdpi.com/article/10.3390/ijms222111611/s1.
