# Supplementary material for: Reduced GFAP Expression in Bergmann Glial Cells in the Cerebellum of Sigma-1 Receptor Knockout Mice Determines the Neurobehavioral Outcomes after Traumatic Brain Injury
Source: Int J Mol Sci. 2021 Oct 27;22(21):11611. doi: 10.3390/ijms222111611 (PMC8584110; doi:10.3390/ijms222111611)
Supplement: Supplementary file 1 [file ijms-22-11611-s001.zip › ijms-1381460-supplementary.pdf]

Supplementary materials

Table S1. Spontaneous alternations (%) in the y-maze test at 1, 3, 6, 9 and 12 months after TBI.

Time, months	1	3	6	9	12
WT sham	60 ± 3	46 ± 4	49 ± 2	64± 4	48 ± 4
WT TBI	55 ± 4	61 ± 3	54 ± 4	61 ± 4	50 ± 3
S1R-/- sham	53 ± 2	56 ± 2	49 ± 3	56 ± 4	55 ± 5
S1R-/- TBI	53 ± 3	55 ± 5	46 ±5	48 ± 3	45 ± 5

All values are presented as means ± SEM. *p* values for differences between groups were calculated using RM two-way ANOVA followed by Fisher's LSD test. WT sham *n*=9, WT TBI *n*=7–10, S1R-/- sham *n*=5–8, S1R-/- TBI *n*=7–10.

Table S2. Time spent in open quadrants of the elevated zero maze at 1, 8, and 12 months after TBI.

Time, months	1	3	8
WT sham	98.79 ± 11.96	130.46 ± 11.68	120.51 ± 8.23
WT TBI	106.86 ± 8.76	129.47 ± 13.85	125.17 ± 17.57
S1R-/- sham	60.08 ± 10.05^	90.38 ± 15.18	112.63 ± 10.28
S1R-/- TBI	82.81 ± 9.48	76.13 ± 12.97	92.15 ±5.40*

All values are presented as means ± SEM. *p* values for differences between groups were calculated using RM two-way ANOVA followed by Fisher's LSD test: * $p < 0.05$ WT TBI vs. S1R-/- TBI, ^ $p < 0.05$ WT sham vs. S1R-/- sham. WT sham *n* = 9, WT TBI *n*=7–10, S1R-/- sham *n*=5–8, S1R-/- TBI *n*=7–10.

Table S3. The total distance travelled (cm) in an open-field apparatus at 7 days and 3, 6, 9 and 12 months after TBI.

Time	Days	Months			
	7	3	6	9	12
WT sham	5738 ± 211	5569 ± 443	5172 ±346	6030 ± 538	5878 ± 594
WT TBI	6207 ± 426	7026 ± 600	6214 ± 698	7299 ± 711	6819 ± 601
S1R-/- sham	5811 ± 374	6087 ± 440	5884 ± 703	5041 ± 455	6319 ± 521
S1R-/- TBI	5558 ± 162	6143 ± 280	5651 ± 124	5599 ± 535	5551 ± 444

All values are presented as means ± SEM. *p* values for differences between groups were calculated using RM two-way ANOVA followed by Fisher's LSD test. WT sham *n*=9, WT TBI *n*=7–10, S1R-/- sham *n*=5–8, S1R-/- TBI *n*=7–10.

Table S4. Food (mg/kg body weight) and water (ml/kg body weight) consumption and voluntary activity (running wheel rotations) at 72 h 1, 3 and 6 months after TBI.

Time, months	1				3				6			
	Food	Drink	Activity		Food	Drink	Activity		Food	Drink	Activity	
			Dark	Light			Dark	Light			Dark	Light
WT sham	0.093 ± 0.01	0.112 ± 0.01	3589 ± 755	2156 ± 849	0.126 ± 0.01	0.091 ± 0.005	5422 ± 1098	3393 ± 816	0.098 ± 0.01	0.070 ± 0.01	3874 ± 488	1772 ± 442
WT TBI	0.101 ± 0.01	0.132 ± 0.01	6409 ± 1312	3913 ± 1067	0.111 ± 0.01	0.067 ± 0.01	5546 ± 1600	1981 ± 603	0.094 ± 0.01	0.072 ± 0.01	4688 ± 1560	1525 ± 780
S1R-/- sham	0.101 ± 0.01	0.106 ± 0.01	7369 ± 1140	2271 ± 337	0.107 ± 0.01	0.082 ± 0.01	8002 ± 726	2550 ± 704	0.110 ± 0.004	0.094 ± 0.004	5258 ± 851	1676 ± 268
S1R-/- TBI	0.106± 0.01	0.099 ± 0.01	5300 ± 1040	3079 ± 253	0.107 ± 0.01	0.084 ± 0.01	7456 ± 327	1785 ± 388	0.138 ± 0.02	0.101 ± 0.02	6112 ± 1244	2223 ± 217

All values are presented as means ± SEM. *p* values for differences between groups were calculated using RM two-way ANOVA followed by Fisher's LSD test, *n* = 7–8.

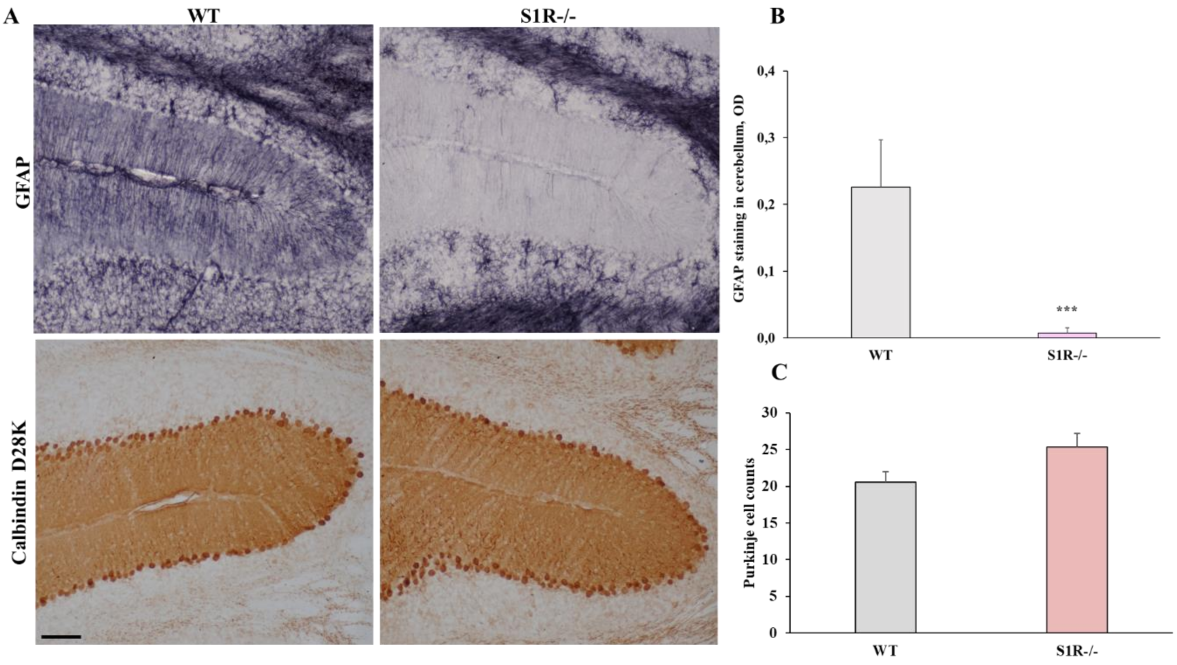

Figure S1. GFAP and Calbindin D28K staining in the cerebellum before TBI. **(A)** Representative images of GFAP and Calbindin D28K staining in the cerebellum (scale bar = 50 μm). **(B)** Optical density (OD) of the intensity of GFAP staining in the molecular layer of the cerebellum in WT and S1R-/- animals. **(C)** Total count of Purkinje cells in the purkinje cell layer of the cerebellum in WT and S1R-/- animals. All values are presented as the means ± SEM. *p* values for differences between groups were calculated using the Mann–Whitney U-test, ($n = 3$): ***$p < 0.001$ WT vs. S1R-/-.

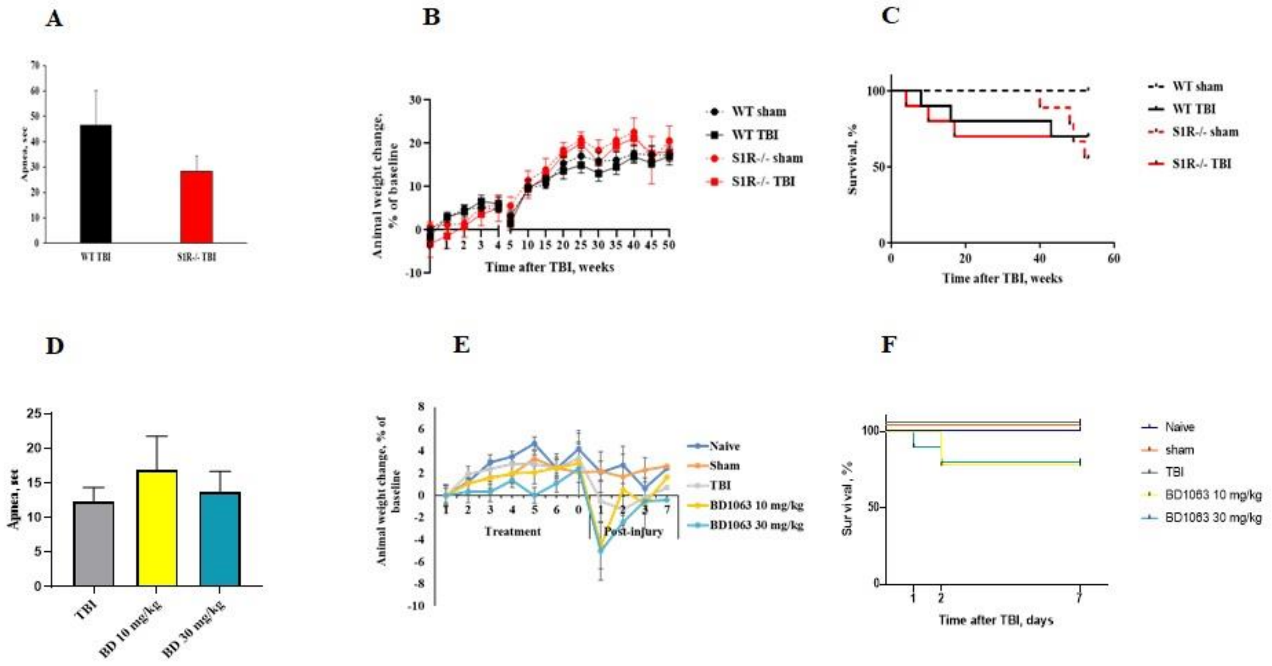

Figure S2. General health monitoring after TBI. **(A)** Apnoea, **(B)** body weight changes and **(C)** mortality in WT and S1R-/- mice after TBI. TBI did not affect general health. **(D)** Apnoea, **(E)** body weight changes and **(F)** mortality in WT mice treated with BD-1063 after TBI. BD-1063 treatment did not influence outcomes after the injury. All values are presented as means ± SEM. *p* values for body weight differences between groups were calculated using two-way ANOVA followed by Fisher's LSD test. Differences in apnoea after TBI between the WT and S1R-/- groups and TBI and BD-1063 10–30 mg/kg groups were calculated using the Mann–Whitney U-test and ordinary one-way ANOVA, respectively. Differences in overall mortality rates were calculated using the chi-square test.
